# WHO World Reports on Hearing and Vision: Meeting the Growing Needs of Older Adults

**DOI:** 10.1093/geroni/igaa057.2930

**Published:** 2020-12-16

**Authors:** Carrie Nieman, Bonnielin Swenor, Charlotte Yeh

## Abstract

The World Health Organization’s (WHO) World Report on Vision was released in October 2019 and the World Report on Hearing debuted at the World Health Assembly in May 2020. The Reports recognize the fundamental nature of sensory health in the health and well-being of individuals and societies and outline the significant and growing burden of hearing loss and vision impairment across the life course. Together the efforts call for more affordable, accessible, and integrated care to foster sensory health for all and represent a major opportunity to advance vision and hearing care as public health priorities nationally and internationally. With the largest burden of sensory impairment among older adults, this symposium will focus on applying the findings and recommendations of the Reports to gerontology. The first presentations will provide an overview of the World Reports on Vision and Hearing from members of the core working groups involved in the Reports and discuss them within the context of the WHO’s Decade of Healthy Aging. The second set of presentations will feature the latest findings related to sensory health from the Global Burden of Disease Study. In moving toward action, task sharing is a critical theme that runs throughout the Reports and we will cover the application of task sharing to hearing care as an example of applying public health principles to advance sensory health. As we advocate for improving the lives of older adults, sensory health remains a significant component with great potential for progress and collaboration.

